# Construction and Functionality of a Ceramic Resonant Pressure Sensor for Operation at Elevated Temperatures

**DOI:** 10.3390/s18051423

**Published:** 2018-05-03

**Authors:** Matej Sadl, Andraz Bradesko, Darko Belavic, Andreja Bencan, Barbara Malic, Tadej Rojac

**Affiliations:** 1 Electronic Ceramics Department, Jozef Stefan Institute, Jamova cesta 39, 1000 Ljubljana, Slovenia; andraz.bradesko@ijs.si (A.B.); darko.belavic@ijs.si (D.B.); andreja.bencan@ijs.si (A.B.); barbara.malic@ijs.si (B.M.); tadej.rojac@ijs.si (T.R.); 2 Jozef Stefan International Postgraduate School, Jamova cesta 39, 1000 Ljubljana, Slovenia; 3 Centre of Excellence NAMASTE, Jamova cesta 39, 1000 Ljubljana, Slovenia; 4 HIPOT-RR, Šentpeter 18, 8222 Otočec, Slovenia

**Keywords:** piezoelectric ceramic resonant pressure sensor, bismuth ferrite, high-temperature applications

## Abstract

Piezoelectric ceramic resonant pressure sensors have shown potential as sensing elements for harsh environments, such as elevated temperatures. For operating temperatures exceeding ~250 °C, conventional and widely used Pb(Zr,Ti)O_3_ (PZT) piezoelectrics should be replaced. Here, a ceramic pressure sensor from low-temperature co-fired ceramics (LTCC) was constructed by integrating a piezoelectric actuator made from bismuth ferrite (BiFeO_3_) on a diaphragm. This ferroelectric material was selected because of its high Curie temperature ($T_{C}$ = 825 °C) and as a lead-free piezoelectric extensively investigated for high-temperature applications. In order to construct a sensor with suitable pressure sensitivity, numerical simulations were used to define the optimum construction dimensions. The functionality of the pressure sensor was tested up to 201 °C. The measurements confirmed a pressure sensitivity, i.e., resonance frequency shift of the sensor per unit of pressure, of −8.7 Hz/kPa up to 171 °C. It was suggested that the main reason for the hindered operation at the elevated temperatures could lie in the thermo-mechanical properties of the diaphragm and the adhesive bonding at the actuator-diaphragm interconnection.

## 1. Introduction

Pressure sensors are required in a wide variety of applications and in different environments that cover a broad range of pressures spanning from below ~10^−5^ bar to above a few 100 bars. Not one sensor is universal and it is not possible to cover such a wide pressure range and the fulfill demands required for each specific application. Pressure sensors have so far been constructed in different designs, the simplest and most commonly used is the edge clamped diaphragm, with integrated sensing elements for detection of its deflection caused by the applied pressure [1]. Different working principles for pressure sensors have been developed [2]. According to the method of deflection measurement, i.e., the conversion of diaphragm deflection into a measurable signal (e.g., electric signal), most pressure sensors can be divided into the categories of piezoresistive [3,4,5,6,7,8], capacitive [9,10,11,12], optical [13,14], and resonant [15,16,17,18]. The resonant pressure sensor operates by monitoring the shift of the diaphragm’s resonance frequency in relation to differential pressure. For a small deflection, the frequency shift is proportional to the change of a differential pressure. During the operation of a resonant pressure sensor, its diaphragm must be in vibrating resonant motion, and the resonance frequency needs to be detected. There are few possibilities for excitation of the vibration and detection of the resonance frequency, i.e., the two tasks can be done either separately or by using a single piezoelectric material as an active element. In the latter case, the piezoelectric acts as an actuator and sensor simultaneously, utilizing the converse and direct piezoelectric effects, respectively. The resonance frequency can be detected, for instance, by monitoring the piezoelectric’s impedance.

Most pressure sensors on the market are made via the micromachining of silicon. The main advantages of these sensors are miniaturization and high-volume production. Nowadays, a lot of attention is focused on the development of sensors, which can withstand demanding conditions under harsh environments. A sensor that operates in harsh environments must be stable under higher temperatures, pressures, mechanical stress, and under the influence of moisture or reactive chemicals. One of the possibilities to design a robust and reliable sensor is to use ceramic materials. A common way to accomplish this is to use low-temperature co-fired ceramics (LTCC) and thick-film technology. LTCC is resistant against heat, corrosion, high pressures, and mechanical wear. The LTCC technology can be used to design three-dimensional (3D) structures of various shapes in meso-sized range (total sizes up to a few cm) and fast prototyping at low price [19].

During the selection of an appropriate piezoelectric material for a given application, the decision most often falls on lead zirconate titanate (Pb(Zr,Ti)O_3_ or PZT), which is known as a high performance ferroelectric [20,21]. Because of its excellent, reliable and versatile piezoelectric properties, PZT is a material of choice for pressure transducers. There are, however, two limitations related to PZT. First, it contains toxic lead and, therefore, should be replaced by environmentally safer materials [20]. Second, its Curie temperature of ~350 °C (which depends on the specific composition and dopants), limits its use at elevated temperatures. Not only is piezoelectric activity lost above $T_{C}$ due to the transition to the paraelectric (non-piezoelectric) cubic phase [22], the piezoelectric performance of PZT may gradually reduce over time during operation at temperatures approaching $T_{C}$ [23].

To overcome the PZT limitations, an alternative ferroelectric material can be employed instead. Here we have chosen bismuth ferrite (BiFeO_3_ or BFO), which is a lead-free high-temperature ferroelectric with an exceptionally high $T_{C}$ (825 °C) [24,25].

The aim of this paper was to construct a functional ceramic resonant pressure sensor with an integrated BFO piezoelectric that could operate at elevated temperatures. The sensor’s functionality was proven to be sufficient until 171 °C. We thus demonstrate the possibility of using BFO as an active piezoelectric material in LTCC resonant sensors and, most importantly, identify the key features that contribute to the reduced functionality at elevated temperatures. The origins of the pressure sensor’s upper temperature limit of operation are multiple but probably dominated by the thermo-mechanical properties of the LTCC diaphragm and the adhesive (bonding agent) at the actuator-diaphragm interconnection.

## 2. Materials and Methods

### 2.1. Design of the Sensor

**Figure 1:**
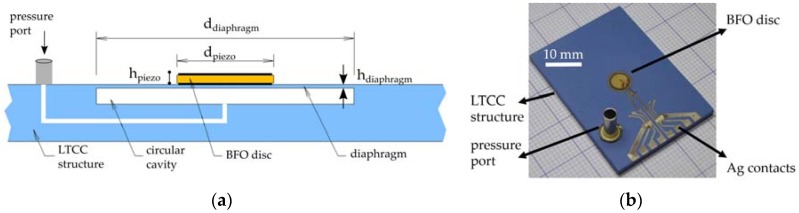
Construction of the pressure sensor; (**a**) schematic cross-section of the sensor’s structure and (**b**) photograph of the actual device. In Figure 1a, the diameter of the diaphragm and BFO disc is marked as d_diapragm_ and d_piezo_, respectively; the thickness of the diaphragm and BFO disc is marked as h_diapragm_ and h_piezo_, respectively.

We have constructed a piezoelectric ceramic resonant pressure sensor designed as an edge-clamped circular diaphragm with an integrated piezoelectric element. The diaphragm is part of a LTCC-based ceramic structure with cavity, channel, pressure port, and thick-film conductors for electrical interconnection. The piezoelectric element in the form of a BFO ceramic disc with electrodes on both facets is bonded onto the diaphragm with a high-temperature conductive adhesive. The piezoelectric element acts as an actuator to drive oscillations of the diaphragm and as a sensor to detect the vibration frequency. Figure 1a represents a schematic cross-section of the sensor’s structure, whereas the photograph of the actual sensor device is shown in Figure 1b.

In the stationary state, the resonance frequency for a circular, edge-clamped diaphragm depends on geometry and material properties, including thermo-mechanical properties. For a particular design of a resonant sensor in the operation mode, the resonance frequency depends on applied pressure and temperature [15]. Our objective was to design a sensor with high pressure sensitivity *S* (Hz/kPa), which is in a resonant pressure sensor defined as the resonance frequency shift ∆f (Hz) for a given pressure change ∆p (kPa).

(1) $$S\;=\;\frac{∆f}{∆p}$$

To investigate the influence of various geometrical parameters on resonance frequency behavior under pressure, we constructed a finite-element model of the pressure sensor using COMSOL Multiphysics software. The details about the numerical simulations are presented in Appendix A. The results of the numerical simulations confirmed that the sensitivity of the pressure sensor increases with (i) an increasing diameter/thickness ratio of the diaphragm and with (ii) decreasing dimensions of the piezoelectric disc (i.e., thickness and diameter in range of 0.10–0.20 mm and 2–10 mm, respectively). However, with a decreasing diameter of the piezoelectric disc, the capacitance of the sensor decreases quadratically. Note that the resonance of the diaphragm is monitored via measuring the impedance of the attached piezoelectric. Thus, choosing the optimal diameter of the piezoelectric disc is a careful interplay between ensuring a measurable impedance and having a sufficient sensitivity of the pressure sensor. The diameter of the piezoelectric of ~5 mm was chosen to be the optimal tradeoff between the appropriate sensitivity and impedance signal.

Besides the results of numerical modeling, the manufacturing limitations (LTCC technology and thinning of ceramics) have also been considered in order to construct the sensor with optimal dimensions and are shown in Table 1. The lower limit in fabrication of the diaphragm with LTCC technology was 0.20 mm, due to the commercial green tape single layer thickness. The LTCC diaphragm with diameter higher than 16.0 mm experienced a complete deformation during firing. For the fabrication of piezoelectric discs, the BFO ceramic pellets were thinned up to the thickness of 0.20 mm, since thinner discs are impractically fragile.

### 2.2. Fabrication

For the construction of the basic three-dimensional (3D) structure (i.e., the diaphragm and the surrounded frame) the LTCC technology was employed. Eight layers of LTCC tape (SK47, KEKO Equipment, Zuzemberk, Slovenia) were shaped by laser patterning and laminated in a multilayer structure at a temperature of 70 °C and compaction pressure of 20 MPa. To avoid a possible deformation of the 3D structure (especially of a relatively thin diaphragm due to a relatively wide cavity) during the lamination, the LTCC structure was laminated separately in two steps. After the final lamination, the LTCC laminate structures were fired for a total of 5 h using a temperature profile consisting of an organic burnout for 1 h at a temperature of 450 °C, followed with 20 min firing at a peak temperature of 875 °C [4]. The silver (Ag)-based thick-film paste (LF171, DuPont, Wilmington, DE, USA) was used for fabricating conductive layers on LTCC structures. The paste was screen-printed on pre-fired LTCC structures, and then fired for 1 h using a temperature profile with 10 min at a peak temperature of 850 °C.

The BFO ceramic pellet was prepared by the solid-state synthesis. First, high purity starting powders Bi_2_O_3_ (99.999%, Alfa Aesar, Karlsruhe, Germany), Fe_2_O_3_ (99.998%, Alfa Aesar, Karlsruhe, Germany), and Co_3_O_4_ (99%, Alfa Aesar, Karlsruhe, Germany) were pre-milled using an identical procedure as described in [26]. The powder mixture was weighted from Bi_2_O_3_ and Fe_2_O_3_ in a molar ratio of 1:1, and a small amount of 0.1 wt% of Co (in the form of Co_3_O_4_) was added to the mixture to reduce the typically elevated electrical conductivity of BFO [27]. Next, the powder mixture was homogenized as described in [26]. The pellets were uniaxially pressed at 150 MPa from the powder mixture and reactively sintered at 780 °C for 4 h with 10 K/min of heating and cooling rates, identical to the process found in [27]. The relative density of the as-sintered pellets (calculated from the dimensions and the mass) was 93%, and according to X-ray powder diffraction, a phase pure BFO was obtained. The pellets, with a diameter of 4.93 mm, were first cut and then ground to the thickness of 0.20 mm. Approximately 100 nm thick and 4 mm in diameter wide Au/Cr electrodes were sputtered on top and bottom surfaces of BFO pellets. To piezoelectrically activate BFO, the pellets were poled with a DC electric field of 100 kV/cm, which was applied on the samples for 15 min, while they were immersed in a silicone oil at room temperature. After the poling procedure, the BFO pellets exhibited a piezoelectric activity ($d_{33}$) of 40 pC/N.

The sensor was completed after the integration of discrete components (the piezoelectric element and the metal pressure port) onto the LTCC structure. Both components were bonded by gluing with a high-temperature two-component silver-based conductive epoxy adhesive (Duralco 124, Cotronics Corp., Brooklyn, NY, USA), which was cured for 4 h at 121 °C. The top electrode of the piezoelectric element was electrically connected with the conductive pad on the LTCC structure through a copper wire (0.08 mm of diameter), which was also glued at its ends.

### 2.3. Measurement Details

**Figure 2:**
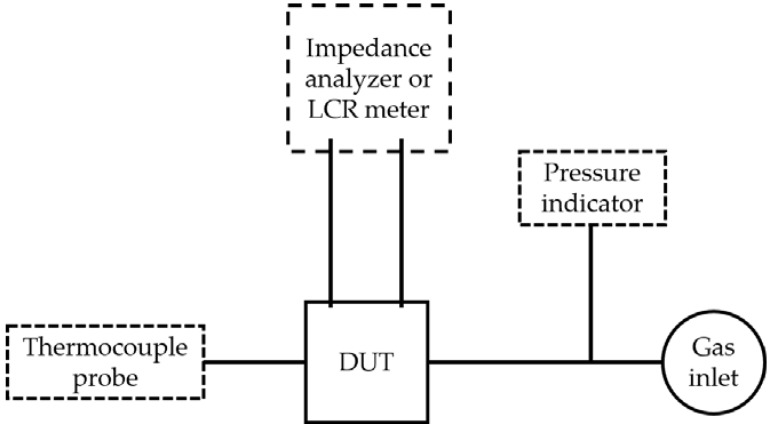
A sketch of the measurement set-up. The device under test (DUT), i.e., the sensor or free-standing BFO piezoelectric disc is electrically connected with an impedance analyzer or LCR meter. Temperature (*T*) and differential pressure (*p*) are monitored by the thermocouple probe and pressure indicator, respectively.

**Figure 3:**
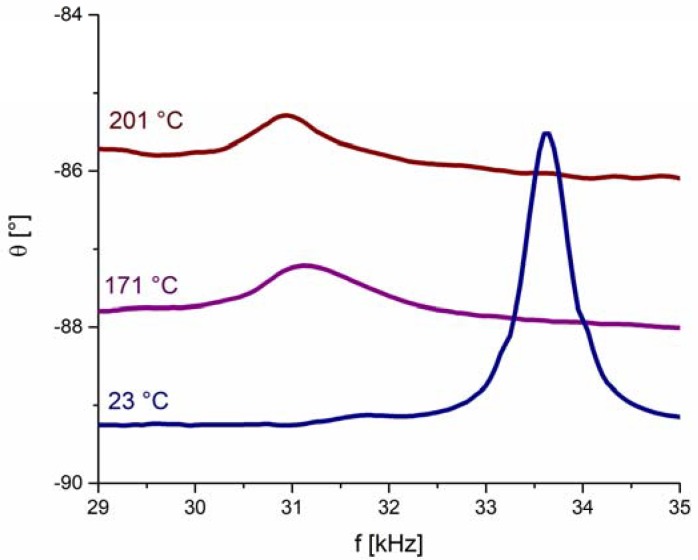
Phase angle *θ* as a function of frequency *f* for the resonant sensor measured at three different temperatures. The density of measured points is 20 kHz^−1^.

**Figure 4:**
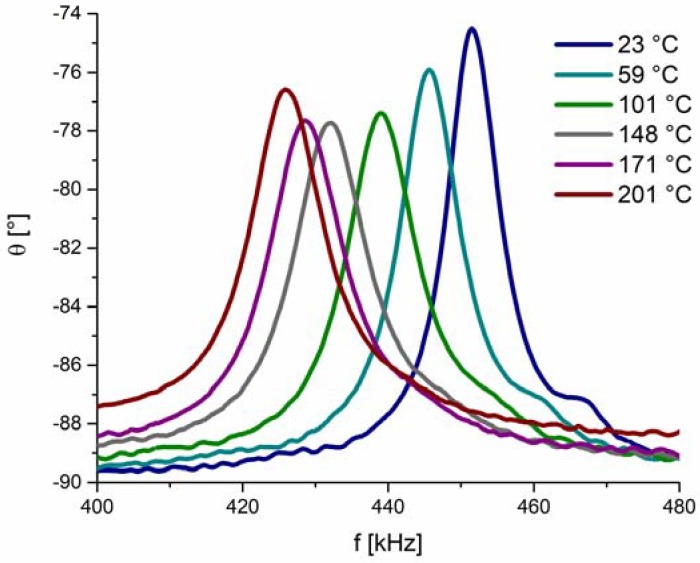
Phase angle *θ* as a function of frequency *f* for the free-standing BFO piezoelectric disc measured at six different temperatures. The density of measured points is 4 kHz^−1^.

**Figure 5:**
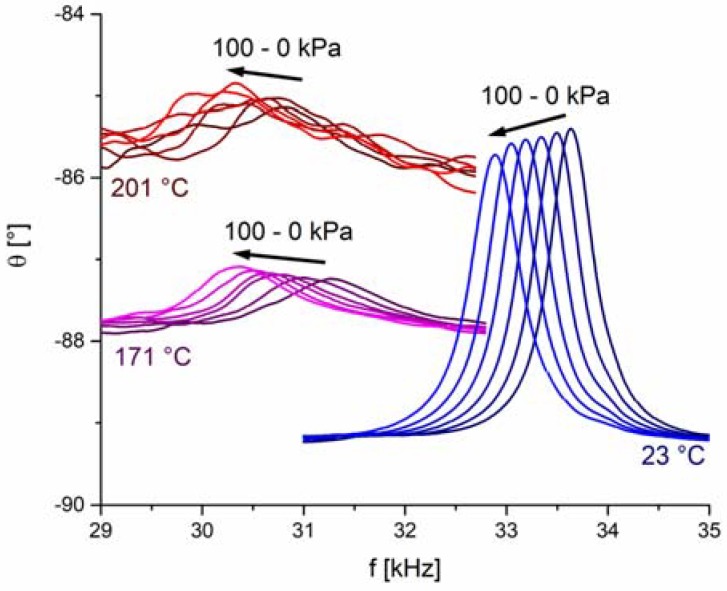
Phase angle *θ* as a function of frequency *f* for the sensor measured at three different temperatures. At each temperature, the measurement was performed by varying the differential pressures (0 kPa, 20 kPa, 40 kPa, 60 kPa, 80 kPa, 100 kPa). The arrows indicate curves for increasing differential pressure. The density of measured points is 100 kHz^−1^.

**Figure A1:**
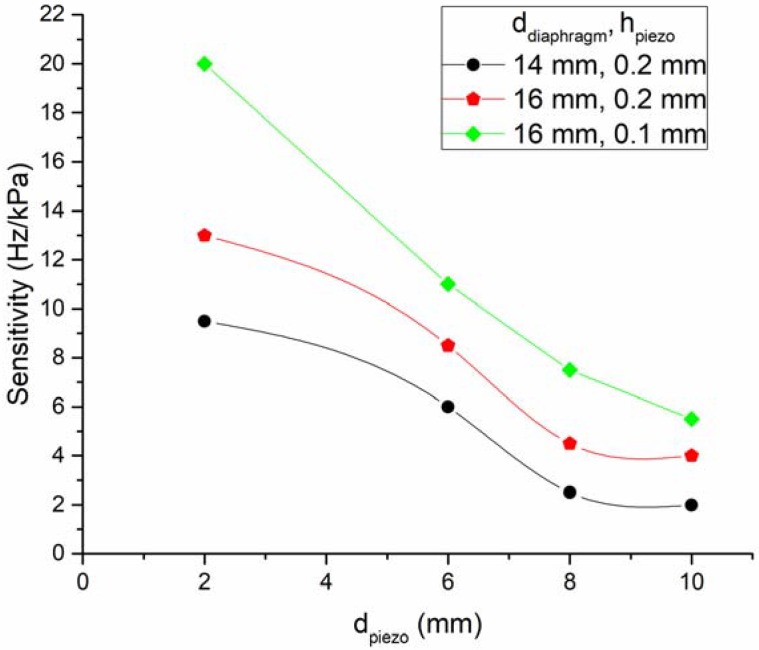
Pressure sensitivity as a function of diameter of the piezoelectric disc (d_piezo_) with variation of other characteristic dimensions, i.e., diameter of the diaphragm (d_diaphragm_) and thickness of the piezoelectric (h_piezo_).

**Figure A2:**
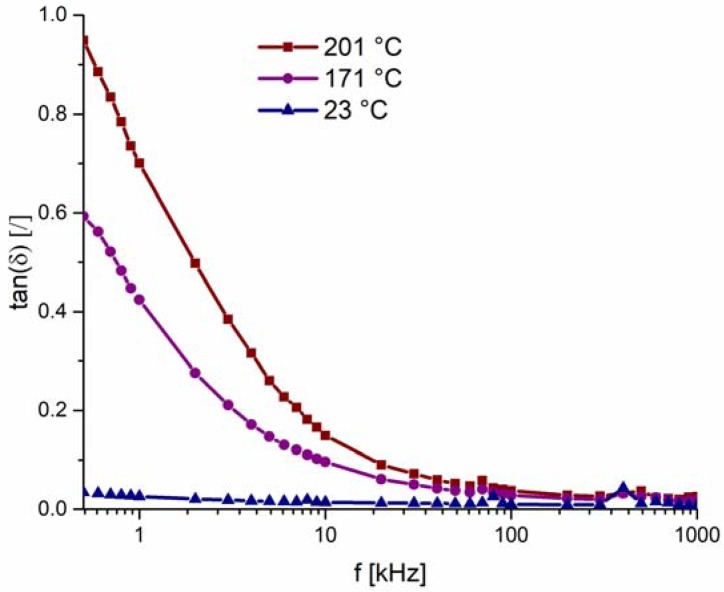
*Tan δ* of the sensor as a function of frequency measured at different temperatures. With increasing temperature, the dielectric losses increase exponentially as the driving frequency is reduced. This low-frequency dispersion in *tan δ* is related to the thermally induced increased leakage current of the BFO driving disc.

The sensor’s functionality was evaluated with impedance measurements (see Figure 2) at the applied differential pressures (ranging from 0 kPa to 100 kPa, with a 20 kPa step) and at three temperatures (23 °C, 171 °C, and 201 °C, with the controlled accuracies of ±1 °C, ±2 °C, and ±3 °C, respectively). The impedance measurements were carried out with the impedance analyzer (4192A LF, Hewlett Packard, Tokyo, Japan) at a frequency range of 29–35 kHz and with the precision LCR meter (4284A LF, Hewlett Packard, Tokyo, Japan) at a frequency range of 5 × 10^2^–10^6^ Hz. During both measurements, the sensor (the device under test–DUT) was placed on a hot plate (REC Digital Ceramic Hot Plate, VELP Scientifica, Usmate, Italy). To apply a differential pressure, a small plastic tube was used to transfer N_2_ gas over the pressure port into the cavity under the diaphragm. The sensor’s temperature was measured with a surface K-type thermocouple probe (TPK-04, Tecpel, New Taipei City, Taiwan) positioned on the upper surface of the sensor’s rigid structure (in the vicinity of the diaphragm). The applied pressure was evaluated with a digital pressure indicator (PM, Heise^®^, Stratford, CT, USA). Another set of impedance measurements was carried out on free-standing BFO piezoelectric discs (DUT) with the impedance analyzer (4192A LF, Hewlett Packard, Tokyo, Japan) at frequency range 400–480 kHz. The sample was positioned in a tube furnace equipped with a K-type thermocouple. The measurements were performed at temperatures 23 °C, 59 °C, 101 °C, 148 °C, 171 °C, and 201 °C (with the controlled accuracy of ±1 °C). During all impedance measurements the root-mean-square voltage ($U_{RMS}$) of 1 V was applied. The curves in Figure 3, Figure 4 and Figure 5, Figure A1, and Figure A2 are plotted by a method using a line fit between the two adjacent measured points. In order to extract the resonance frequencies, we have fitted the resonance peaks (see Figure 5) with a Gaussian curve. The maximum of the fit represents the resonance frequency.

The depth profile of the sensor’s diaphragm was determined with a stylus profilometer (DektakXT, Bruker, Tucson, AZ, USA).

## 3. Results and Discussion

Each vibrating system has its specific natural resonance frequencies, depending on the construction (dimensions, mechanical, electrical and electromechanical properties) of the diaphragm and the piezoelectric disc. A common way to evaluate the sensor’s numerous resonance frequencies is to measure impedance ($Z_{0}$) and current-voltage phase angle (θ). When plotting θ vs. frequency (*f*), the peaks represent the frequencies of different resonant modes. Due to the observed low $Z_{0}$ signal of the sensor, the sensor resonance behavior is further analyzed using the phase angle peaks.

We have monitored the sensor’s resonance frequency at elevated temperatures, which is positioned in a narrow frequency region, i.e., between 29 kHz and 35 kHz (Figure 3). This particular resonance frequency mode was chosen because it is free of any parasitic effects, such as resonance overlapping. Figure 3 shows θ-versus-*f* curves with the corresponding resonance peaks measured at three different temperatures. From these results we first notice an increase of the background signal with increasing temperature, which can be attributed to the increase of electrical conductivity (leakage current) in the BFO disc (see Appendix B for details). The second obvious observation is that the increase of the operating temperature shifts the θ peak to lower frequencies and increases significantly the peak width, which is inversely proportional to the quality (*Q*) factor [28]. Such a decrease in *Q* factor limits the sensing of the resonance peaks and therefore poses an upper temperature limit in operation of the pressure sensor.

As we will soon see, factors contributing to the reduction of *Q* may have different origins and may work concurrently. The first simple question is whether the degradation of the *Q* factor of the sensor is predominantly related to the piezoelectric BFO disc itself or has origins other than those related to BFO disc. To elucidate this point, we performed similar impedance measurements at elevated temperatures as with the sensor but this time using a free-standing BFO piezoelectric disc (not integrated as part of the sensor). The results in Figure 4 show the radial resonance frequencies of the cylindrical BFO pellet measured at elevated temperatures and in the frequency range 400–480 kHz. The data revealed broadening of the peaks with increasing temperature, suggesting a reduction in *Q* factor. However, this broadening occurs to a much lesser extent than that observed in the same temperature range (23–201 °C) for the sensor (Figure 3). Therefore, by comparing the resonance behavior of the sensor (Figure 3) with that of the free-standing piezoelectric disc (Figure 4), we can conclude that the *Q* factor reduction is more pronounced in the sensor. This indicates that the reduction of the sensor’s *Q* factor is not only related to the intrinsic nature of the active BFO element, i.e., to the material properties of BFO piezoelectric disc, but has other origins that dominate the *Q* factor of the sensor. These other origins may include, for example, the thermo-mechanical properties of the LTCC diaphragm and the adhesive (bonding agent) at the actuator-diaphragm interconnection. Namely, elevated temperatures lead to an increase in the loss modulus (or mechanical loss factor) of the LTCC diaphragm and the epoxy adhesive, which may enhance the damping of the piezoelectric vibrations and consequently contribute to the lowered *Q* factor of the sensor.

As a next step, we analyze the pressure sensor’s ability to operate at elevated temperatures. The differential pressure (*p*) was added as an additional parameter to the first experiment (Figure 3). By monitoring the resonance frequency shift at various differential pressures, we can evaluate the sensor’s pressure sensitivity. Figure 5 shows the phase angle *θ* of the sensor in the frequency range between 29 kHz and 35 kHz for six differential pressures, which were varied at each measurement temperature. The data show a clear shift of the resonance peak to lower frequencies as the pressure increases from 0 kPa to 100 kPa. We found out that above ~170 °C (see example data for 201 °C) the resonance frequency cannot be reliably detected because of the reduced *Q* factor and increased noise level. We can thus conclude that the designed pressure sensor is functional up to a temperature of ~170 °C. Due to a decrease of the sensor’s functionality, no testing was performed above 201 °C.

**Figure 6:**
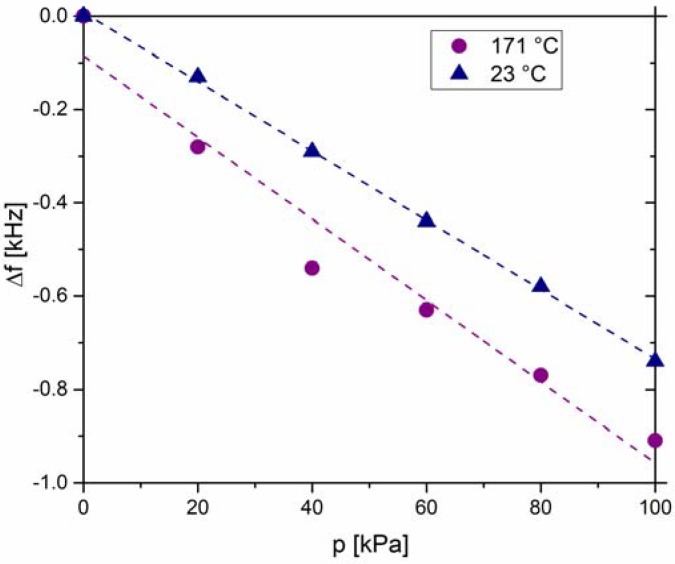
Resonance frequency shift (∆*f*) plotted with respect to the differential pressure (*p*) at 23 °C and 171 °C. The frequency shift was calculated accordingly to the starting point which is the resonance frequency at 0 kPa.

The resonance frequencies, as a function of differential pressure at 23 °C and 171 °C, obtained from Figure 5, are plotted in Figure 6. The results show a near-linear relationship between the resonance frequency and the differential pressure. Note that the linear regression *R*^2^ factor drops from 0.999 to 0.961, when the temperature is increased from 23 °C to 171 °C. The pressure sensitivity calculated from the slope of the curves is given in Table 2 and reaches −7.4 Hz/kPa and −8.7 Hz/kPa at 23 °C and 171 °C, respectively. The sensitivity thus increases for ~18% with increasing temperature. We note that these sensitivity values cannot be straightforwardly compared with those of other resonant pressure sensors because the sensitivity is strongly affected by the particular sensor design, and therefore, the pressure sensitivity of different resonant sensors can vary within a range of three orders of magnitude (from ~1 Hz/kPa to ~1000 Hz/kPa) [15,16,18,29,30,31].

**Figure 7:**
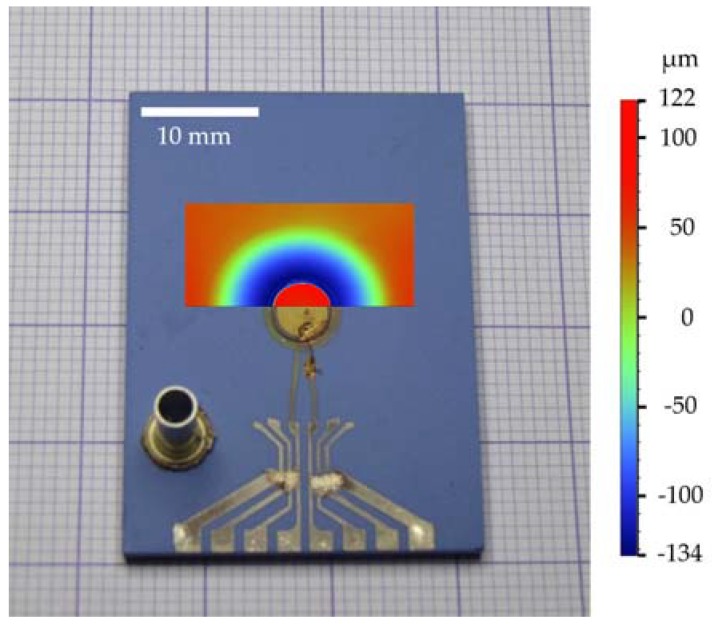
Depth map of the sensor’s diaphragm overlaid on a photograph of the sensor. The color scale bar revealing depth is shown on the right side of the photograph. Negative depth values refer to downward in-plane curvature, while positive values to upward in-plane curvature.

We note in Figure 5 that the resonance frequency decreases with increasing differential pressure, or in other words, the sensitivity of the pressure sensor is negative. An opposite behavior, where the sensitivity was positive, has previously been observed in the literature [15,16,29]. The reason for such an anomalous behavior in our experiment may lie in the construction details (i.e., the sagging [32,33]) of the sintered LTCC diaphragm. The diaphragm is actually bent down (toward the circular cavity, see Figure 1a), which was confirmed with depth mapping (see Figure 7). This curvature indicates that the diaphragm is under residual stress, which builds-up during the firing and cooling of the LTCC structure. When pressure is applied on the convex surface, the residual stresses are reduced and the resonance is therefore shifted to lower frequencies [34,35]. A description of the relations between a diaphragm’s stresses and resonance frequency can be found in [35], where it was shown that the stress conditions in the diaphragm mediate the frequency shift.

In addition to differential pressure, Figure 3 and Figure 5 show that the resonance frequency also shifts due to temperature change. This can be explained with the temperature dependence of BFO’s properties in the piezoelectric disc (Figure 5) and also in a similar way as for the negative pressure sensitivity. The diaphragm expands with increasing temperature, causing stress relaxation in the diaphragm, which shifts the resonance frequency to lower values.

## 4. Summary and Conclusions

A piezoelectric ceramic resonant pressure sensor was designed and constructed for potential use in harsh environments, particular at high temperatures. An LTCC edge clamped circular diaphragm was designed to act as a vibrating body, which was actuated with an integrated piezoelectric element in the form of a BFO ceramic disc. BFO has been chosen because of its high Curie temperature (825 °C) which makes it a promising candidate for high-temperature applications.

The functionality of the pressure sensor was tested at temperatures up to 201 °C in a range of a differential pressure between 0 kPa and 100 kPa. The measurements confirmed the pressure sensitivity of −8.7 Hz/kPa at the maximum temperature of 171 °C. At higher temperatures, the sensing ability was suppressed due to the increased noise level in the measured impedance, namely, current-voltage phase angle *θ*, and due to the reduction of *Q* factor of the exploited sensor’s resonance mode. It was suggested that the origins of the *Q* factor reduction are multiple but probably dominated by the thermo-mechanical properties of the LTCC diaphragm and the adhesive (bonding agent) at the actuator-diaphragm interconnection. Therefore, to expand the upper operation temperature of the sensor, further work would have to be focused on the optimization of the thermo-mechanical properties of the sensor’s diaphragm and the adhesive.

We found that the fabrication of the sensor’s LTCC structure results in a slightly curved and pre-stressed diaphragm at room temperature. The residual stress in the diaphragm relaxes with the applied pressure, which ought to be responsible for the observed negative pressure sensitivity.

## Figures and Tables

**Table 1:** Dimensions of the constructed piezoelectric pressure sensor, which have been chosen based on the results of the numerical modelling and given manufacturing limitations.

Parts of a Sensor	Dimensions (mm)
d_diapragm_	16.0
d_piezo_	4.93
h_diapragm_	0.20
h_piezo_	0.20

**Table 2:** Sensitivity (*S*) and linear regression factor *R*^2^ for measurements at 23 °C and 171 °C. The values were evaluated after fitting the frequency shift data with the linear curve (see Figure 6).

*T* (°C)	*R*^2^	*S* (Hz/kPa)
23	0.999	−7.4
171	0.961	−8.7

## Appendix A

Numerical simulations were used to study the pressure sensitivity as a function of the characteristic dimensions of the sensor, i.e., diameter and thickness of the piezoelectric disc and diameter of the diaphragm.

A numerical model of a piezoelectric resonant pressure sensor was built according to Figure 1a. The geometry of the sensor system was modelled in 3D. In the actual construction of the sensor the piezoelectric disc is attached with an adhesive at the center of the circular LTCC diaphragm. The movement of the diaphragm was constrained at its edges by clamping it on a rigid structure. The characteristic dimensions were constrained as follows. The diaphragm diameter was set at either 14 mm or 16 mm at fixed thickness of 0.20 mm. The thickness of the integrated piezoelectric disc was set at either 0.10 mm or 0.20 mm with diameter dimensions ranging from 2 mm to 10 mm.

Material properties of LTCC and the adhesive [36] are listed in Table A1. Please note, that the material properties of the piezoelectric element were described by a Pb(Zr,Ti)O_3_ (PZT) (commercial PZT-5H). This is due to the well-known material properties of the PZT-5H material. The piezoelectric and stiffness matrix of PZT were taken from literature [37].

**Table A1:** Material properties of LTCC and adhesive.

Material	Density (kg/m^3^)	Young’s Modulus (GPa)	Poisson Ratio
LTCC	3100	110	0.17
Adhesive	1600	5.5	0.4

The results of the simulations shown in Figure A1 confirm that for a given range of characteristic dimensions the sensitivity of the pressure sensor increases with (i) increasing diameter/thickness ratio of the diaphragm and with (ii) decreasing dimensions (i.e., thickness and diameter) of the piezoelectric disc.

## Appendix B

To find out whether the increase of the background level of *θ* with increasing temperature, shown in Figure 3, is indeed related to increased electrical conductivity of BFO, we measured the dielectric losses (*tan δ*) of the sensor in a wider frequency range (from 0.5 kHz to 1 MHz). Note that *δ* denotes the charge-voltage phase angle, which is straightforwardly related to the current-voltage phase angle *θ*. Figure A2 reveals a strong frequency dispersion of *tan δ* in the low frequencies range becoming more apparent at elevated temperatures. This strong frequency dispersion of *tan δ* with frequency may be understood by considering the contribution of the DC electrical conductivity to *tan δ*:

(A1) $$tan\;\delta \;=\;\frac{\sigma_{DC}}{\varepsilon^{\text{’}}\;\cdot \;\omega }$$

where $\sigma_{DC}$, $\varepsilon^{\text{’}}$, and ω represent specific DC conductivity (Ω^−1^ m^−1^), real part of permittivity (F m^−1^) and angular frequency (s^−1^) respectively [38]. As can be seen in Equation A1, *tan δ* becomes proportional to $\sigma_{DC}/\omega$; therefore, as the conductivity $\sigma_{\mathrm{DC}}$ is increased at elevated temperatures tan *δ* will show a strong 1/ω dispersion (i.e., an abrupt increase at low frequencies), which is effectively seen in Figure A2.
